# CD4 ^+^ T‐cell responses directed towards A/H5N1‐derived haemagglutinin peptides increase in patients with seasonal influenza A virus infection

**DOI:** 10.1002/cti2.70118

**Published:** 2026-08-02

**Authors:** Lilith F Allen, Louise C Rowntree, Mitchell Jenzen, Ruth R Hagen, Nathan P Croft, Fiona James, Genevieve E Martin, Anthony W Purcell, Patricia T Illing, Steven Y C Tong, Allen C Cheng, Tom C Kotsimbos, Jason A Trubiano, Katherine Kedzierska, Thi H O Nguyen

**Affiliations:** ^1^ Department of Microbiology and Immunology The University of Melbourne, at the Peter Doherty Institute for Infection and Immunity Melbourne VIC Australia; ^2^ Department of Biochemistry and Molecular Biology, Immunity Program, Biomedicine Discovery Institute Monash University Clayton VIC Australia; ^3^ Department of Human‐Centred Computing Monash University Clayton VIC Australia; ^4^ Department of Infectious Diseases and Immunology Austin Health Heidelberg VIC Australia; ^5^ Victorian Infectious Diseases Service, The Royal Melbourne Hospital Peter Doherty Institute for Infection and Immunity Melbourne VIC Australia; ^6^ Department of Infectious Diseases University of Melbourne, at the Peter Doherty Institute for Infection and Immunity Melbourne VIC Australia; ^7^ School of Public Health and Preventive Medicine Monash University Melbourne VIC Australia; ^8^ Monash Infectious Diseases, Monash Health and School of Clinical Sciences Monash University Clayton VIC Australia; ^9^ Department of Respiratory Medicine The Alfred Hospital Melbourne VIC Australia; ^10^ Department of Medicine, Central Clinical School, The Alfred Hospital Monash University Melbourne VIC Australia; ^11^ Department of Infectious Diseases Peter MacCallum Cancer Centre Melbourne VIC Australia; ^12^ National Centre for Infections in Cancer Peter MacCallum Cancer Centre Melbourne VIC Australia; ^13^ Center for Influenza Disease and Emergence Response (CIDER) Athens GA USA

**Keywords:** CD4^+^ T cells, H5N1, immunology, influenza virus, pandemic influenza, T cells

## Abstract

**Objectives:**

Recent outbreak of influenza A/H5N1 viral infections in birds and mammals worldwide prompted renewed concerns over an emerging H5N1‐related influenza pandemic in the generally immunologically‐naive global population. Cross‐reactive CD4^+^ T cells between A/H1N1 and A/H5N1 subtypes targeting internal and surface influenza proteins have been identified in humans, indicating some protection against severe and fatal A/H5N1 infection.

**Methods:**

To detect T‐cell responses towards H1 and H5 viruses, we stimulated PBMCs from patients hospitalised with seasonal influenza A viruses, alongside healthy individuals, with overlapping peptide pools spanning hemagglutinin (HA) and neuraminidase (NA) of A/Vietnam/1203/2004 (H5N1) and A/New York/18/2009 (H1N1‐pdm09‐like) viruses.

**Results:**

Majority of acutely infected and healthy participants (100% and 75%, respectively) had detectable CD4^+^ and CD8^+^ T‐cell responses towards H1‐HA and H5‐HA, whereas 30–80% of individuals had detectable H5‐NA responses. H1 and H5 HA‐specific CD4^+^ T‐cell responses highly correlated, suggesting some level of cross‐reactivity between T‐cell responses directed against H1 and H5 subtypes. HA‐specific CD4^+^ T‐cell responses were increased in influenza patients compared to healthy participants. We found no differences in H1‐ and H5‐specific T cell responses between those born pre‐ and post‐1968, irrespective of infection status. Sequence alignment of H1 and H5 viruses identified high conservation in the HA2 stalk subunit (81.53%). CD4^+^ T‐cell immunity towards immunodominant HA_306–318_ epitope, restricted by several HLA‐DRB1 molecules, revealed limited cross‐reactivity between Group 1 and Group 2 HA‐subtypes.

**Conclusion:**

We define correlative H1‐ and H5‐specific CD4^+^ T‐cell responses during seasonal influenza A virus infection, likely reflecting some level of H1/H5 T cell cross‐reactivity.

## Introduction

Influenza A remains a significant global health threat, causing a large burden of severe illness, hospitalisation and fatalities annually, especially among individuals with underlying vulnerabilities.^1^ Highly pathogenic avian viruses, including A/H5N1 and A/H7N9, cause sporadic outbreaks in wild and domestic birds and occasionally in humans.^2^ H5N1 Clade 2.3.4.4b is now well‐established in wild bird populations globally (except Australia),^3^ has spilled over into multiple mammalian populations^4^, ^5^ and recently resulted in 71 human cases and two deaths in the United States after an outbreak in dairy cattle.^6^ These cases were predominantly dairy cattle workers with mild infections dominated by conjunctivitis.^7^ Clade 2.3.4.4b, however, poses pandemic potential, especially if it acquires mutations associated with increased mammalian transmission and pathogenicity, one of which, E627K, has already been detected in a severe case in British Colombia.^8^, ^9^ Because of limited neutralising immunity in the general population against H5 influenza subtypes,^10^ the potential for cross‐reactive T cells to provide cross‐strain immunity could partially protect against severe disease in the event of sustained human–human transmission.^11^, ^12^

Cross‐reactive T cells recognising conserved epitopes have been well‐documented for internal, slowly mutating influenza proteins, particularly NP, M1 and PB2.^13^, ^14^, ^15^ However, cross‐reactive T cells targeting the hemagglutinin (HA) and neuraminidase (NA) proteins on the viral surface are less studied, despite the fact that HA‐directed CD4^+^ T‐cell responses have been previously detected in healthy and influenza virus‐infected individuals.^16^, ^17^ The most prominent CD4^+^ T cell HA‐derived influenza epitopes include (i) the A/H3N2‐derived epitope HA_306–318_ PKYVKQNTLKLAT restricted by several HLA‐DRB1 molecules (01:01/04:01/11:01),^18^, ^19^ which has high homology across influenza A subtypes (shown later), and (ii) a slowly mutated sequence from the HA fusion segment, HA_342‐356_ SRGLFGAIAGFIEGG, restricted by HLA‐DRB1*09:01, which is highly conserved between influenza A viral subtypes as well as influenza B viruses.^20^ Within the HA protein, the head region represents the predominant target for neutralising antibodies and is highly mutated even among related strains, while the more highly conserved stem segment shows higher homology with other subtypes, especially among Group 1 HA's, such as H1, H2, and H5.^21^, ^22^

Here, we sought to define HA‐ and NA‐specific T‐cell responses towards seasonal A/H1N1 and A/H5N1 in patients hospitalised with seasonal influenza A viral infections during 2023–2026 and healthy individuals. PBMCs from influenza A patients and healthy individuals, both highly unlikely to be exposed to A/H5N1 viruses, were stimulated with overlapping peptide pools corresponding to the full HA and NA proteins of A/Vietnam/1203/2004 (H5N1) and A/New York/18/2009 (H1N1‐pdm09‐like) viruses, to detect functional cytokine production. At the protein level, sequence alignment of the HA and NA proteins of our A/H5N1 and A/H1N1 sequences revealed lower conservation in the HA protein (62.89%) compared to NA (83.65%). Cross‐reactive CD4^+^ T‐cell responses at the HA‐specific epitope level were next assessed between Group 1 and Group 2 HA subtypes in the context of the HA_306‐318_ epitope, which is predominantly restricted by HLA‐DRB1.

## Results and discussion

### H5N1‐specific CD4 ^+^ T‐cell responses increase during seasonal influenza A virus infection

Patients hospitalised in 2023–2026 with seasonal influenza A infection (*n* = 21) were recruited a median of 4 days post‐symptom onset (range 2–14) in Melbourne, Australia, as previously described.^23^ Influenza A patients (38% female) had a median age of 59 years (range 19–79), while healthy individuals recruited for comparison (*n* = 17, 53% female) also had a median age of 59 years (range 22–82) (Supplementary figure 1a). Summary demographics are provided in Supplementary table 1.

**Figure 1:**
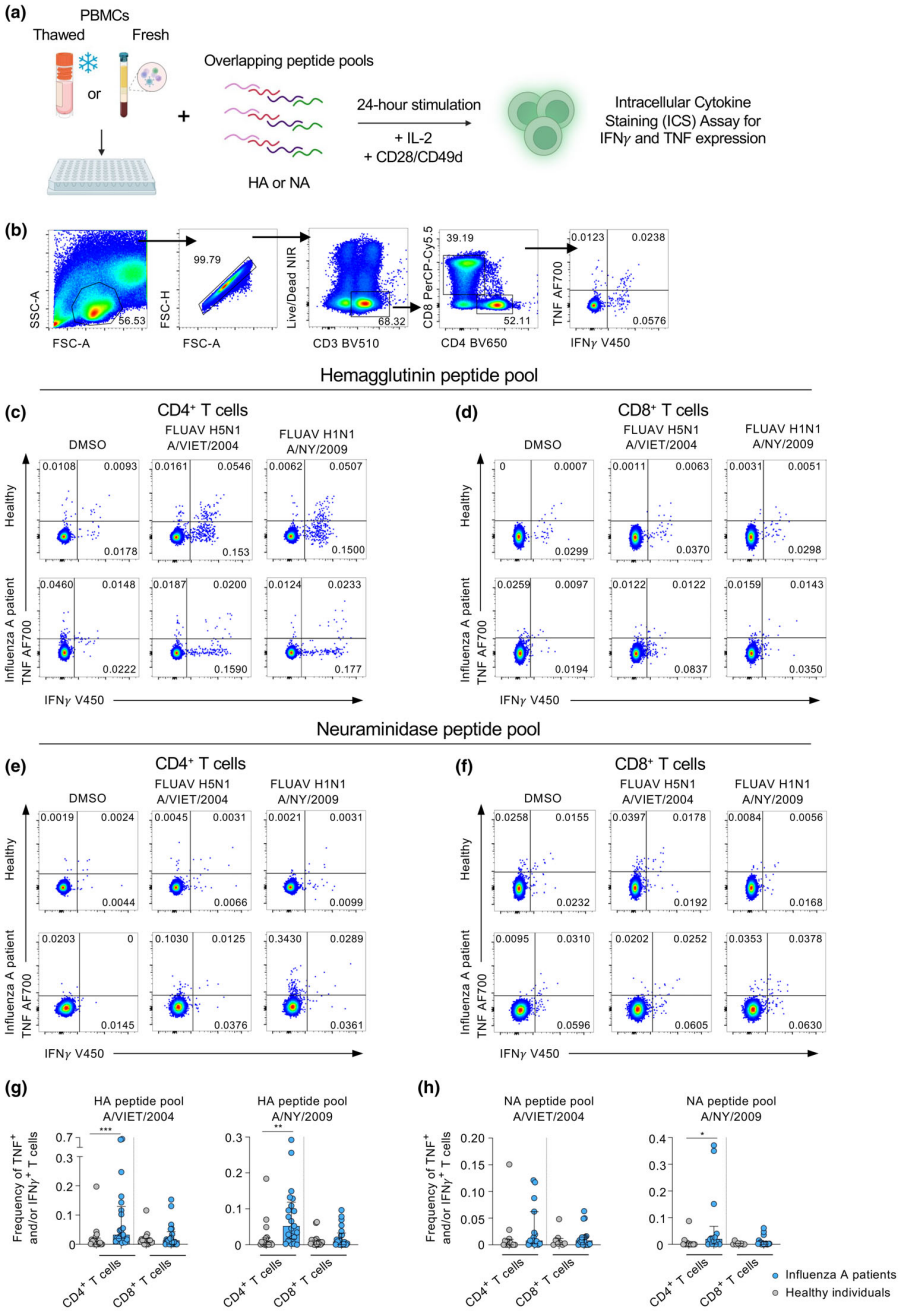
HA‐directed CD4^+^ T‐cell responses increase during influenza virus infection. **(a)** Stimulation of PBMCs from seasonal influenza A infection (*n* = 21) and healthy individuals (*n* = 17) with overlapping peptide pools to assess cytokine responses towards HA and NA **(b)** Gating strategy of intracellular cytokine staining. **(c–f)** Representative intracellular TNF and IFN𝛾 staining of CD4^+^ and CD8^+^ T cells in response to HA and NA overlapping peptide pools. **(g)** Frequency of TNF‐ and/or IFN𝛾‐positive CD4^+^ and CD8^+^ T cells in response to H5 HA A/Viet/2004 and H1 HA A/NY/2009 peptide pool stimulation, with background DMSO subtracted. **(h)** Frequency of TNF‐ and/or IFN𝛾‐expressing CD4^+^ and CD8^+^ T cells in response to N1 NA A/Viet/2004 and N1 NA A/NY/2009 peptide pool stimulation, with background DMSO subtracted. Statistical significance was determined by Mann–Whitney where *P < 0.05, **P < 0.01, and ***P < 0.001. Bars indicate median and IQR.

To evaluate influenza A‐specific T‐cell responses in influenza patients and healthy individuals, PBMCs were stimulated with overlapping peptide arrays covering the entire length of the HA and NA proteins of A/H5N1/Vietnam/1203/2004 (A/VIET/2004) and A/H1N1/New York/18/2009pdm09‐like (A/NY/2009) influenza strains for 24 h and assessed for their IFNγ and TNF responses (Figure 1a and b). Cytokine responses (i.e. TNF^+^ single‐positive, IFNγ^+^ single‐positive and TNF^+^IFNγ^+^ double‐positive T cells) varied between individuals and so total cytokine response was used for subsequent analyses. Robust T‐cell responses to the HA peptide pool of both A/VIET/2004 and A/NY/2009 were detected in both influenza patients and healthy individuals (Figure 1c and d), with all influenza virus‐infected patients demonstrating detectable (response above DMSO) T‐cell responses towards both strains, compared to 75% of healthy individuals. Notably, the HA‐specific CD4^+^ T‐cell responses in influenza virus‐infected patients was higher than in healthy individuals towards both A/VIET/2004 and A/NY/2009 (Figure 1g, *P* = 0.0003 and *P* = 0.0023, respectively), whereas CD8^+^ T‐cell responses were comparable between influenza patients and healthy individuals. Samples were also stimulated with the A/VIET/2004 and A/NY/2009 NA peptide pools, revealing that 60–78% and 33–83% of influenza patients and healthy individuals, respectively, had detectable NA‐specific CD4^+^ and CD8^+^ T‐cell responses (Figure 1e and f). These NA responses directed towards the A/VIET/2004 peptide pool were comparable between groups, while frequencies were lower compared to the HA response (Figure 1h). NA‐specific CD4^+^ T‐cell responses towards the A/NY/2009 NA peptide pool were higher in influenza‐infected patients than in healthy individuals (*P* = 0.0113). Analysis of cytokine responses as a stimulation index, based on fold change above DMSO, confirmed our frequency results which subtracts the DMSO background frequency.

**Figure 2:**
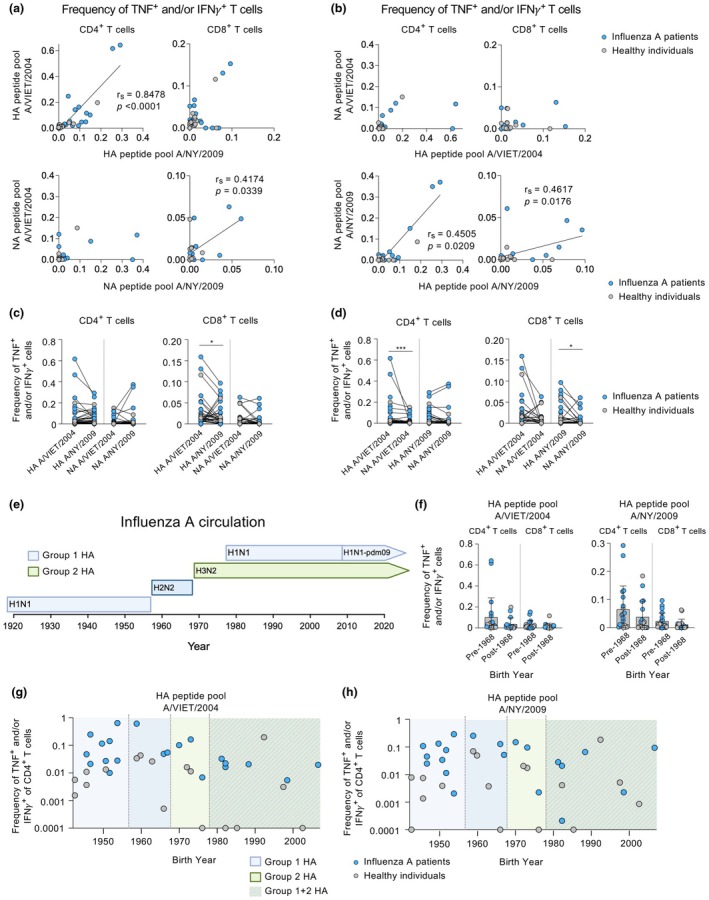
HA‐specific T‐cell responses strongly correlate between Group 1 H1 and H5 strains. Correlations of **(a)** HA and NA cytokine responses between H1 and H5 strains and **(b)** HA and NA responses from the same strain. **(c, d)** Frequency of HA and NA cytokine responses between H1 and H5 peptide pools shown as a paired analysis within individuals. **(e)** Representation of main influenza A subtypes circulating in the last 100 years. **(f)** Frequency of TNF‐ and/or IFN𝛾‐expressing CD4^+^ and CD8^+^ T cells in response to H5 HA A/Viet/2004 and H1 HA A/NY/2009 peptide pool stimulation graphed by those born pre‐ and post‐1968. **(g, h)** Frequency of TNF‐ and/or IFN𝛾‐positive CD4^+^ T cells graphed by birth year and coloured by suspected HA group of first exposure. Data points include patients with seasonal influenza A infection (*n* = 21) and healthy individuals (*n* = 17). Statistical significance was determined by **(a, b)** Spearman r correlation, **(c, d)** Wilcoxon *t*‐test and **(f)** Mann–Whitney where *P < 0.05, and ***P < 0.001. Bars indicate median and IQR.

CD4^+^ T‐cell responses strongly correlated between HA‐H5 A/VIET/2004 and HA‐H1 A/NY/2009 (*r*_*s*_ = 0.8478, *P* < 0.0001, Figure 2a). However, no correlations were observed between H1 and H5 HA‐specific CD8^+^ T cells or between H1 and H5 NA‐specific CD4^+^ T‐cell responses, whereas H1 and H5 NA‐specific CD8^+^ T‐cell responses correlated (*r*_*s*_ = 0.4174, *P* = 0.0339). Our correlative data suggest the presence of H1N1 and H5N1 cross‐reactive T‐cell responses in the HA‐specific CD4^+^ T‐cell response and NA‐specific CD8^+^ T‐cell response, although these associations do not confirm functional cross‐reactivity. CD4^+^ and CD8^+^ T‐cell responses towards the HA and NA peptide pools also correlated for H1N1 A/NY/2009 (*P* = 0.0209 and *P* = 0.0176, respectively, Figure 2b), but not for H5N1 A/VIET/2004.

These findings were supported by paired analysis showing that T‐cell responses towards HA A/VIET/2004 and A/NY/2009 differed within individuals only in the CD8^+^ T‐cell compartment (*P* = 0.0194, Figure 2c), whereas CD4^+^ T‐cell responses were comparable. Similarly, responses towards the NA A/VIET/2004 strain were significantly lower than HA A/VIET/2004 responses in CD4^+^ T cells (*P* = 0.0003, Figure 2d), whereas CD8^+^ T‐cell responses were lower towards the NA A/NY/2009 (*P* = 0.0172, Figure 2d) than HA A/NY/2009.

In support of our findings, CD4^+^ T cells are known to typically dominate the HA epitope‐specific T‐cell response, while CD8^+^ T‐cell epitopes are spread more evenly across external and internal influenza A viral proteins.^24^ Higher frequencies of cytokine‐producing CD4^+^ T cells than CD8^+^ T cells that respond to HA‐derived peptides were demonstrated previously in healthy adult individuals,^12^, ^14^ including in response to avian H5 strains. As HLA types of individual participants can define prominence of influenza‐specific T‐cell responses,^25^ we performed HLA‐typing of study participants. No striking biases in HLA expression between influenza patients and healthy individuals were detected to bias the overall T‐cell response (Supplementary figure 1b and c).

We next investigated HA‐specific T‐cell responses from our influenza patients and healthy individuals in the context of potential childhood hemagglutinin imprinting. Participants born before 1968 were likely to be infected with a group 1 virus (H1N1 or H2N2) as their first influenza exposure before the emergence of H3N2 in 1968 (Figure 2e), as most children have experienced their first influenza virus infection by the age of 3 years.^26^ Participants born between 1968 and 1977 likely experienced the group 2 H3N2 virus as their first encounter, while those born after 1977 were likely exposed to either H1N1 or H3N2 influenza viruses, with H1N1 or H3N2 dominance varying between seasons and across regions. However, we found no significant differences in H1‐ and H5‐specific T‐cell responses between those born pre‐ and post‐1968, irrespective of infection status (Figure 2f–h). Our study shows, for the first time, the presence of H5N1‐reactive CD4^+^ T‐cell responses during acute seasonal influenza A virus (IAV) infections which are higher than those detected in healthy individuals, which suggests that these CD4^+^ T‐cell responses may be influenced by conserved Group 1 HA and NA sequences.

### Sequence conservation between contemporary H1N1 and H5N1 viruses

**Figure 3:**
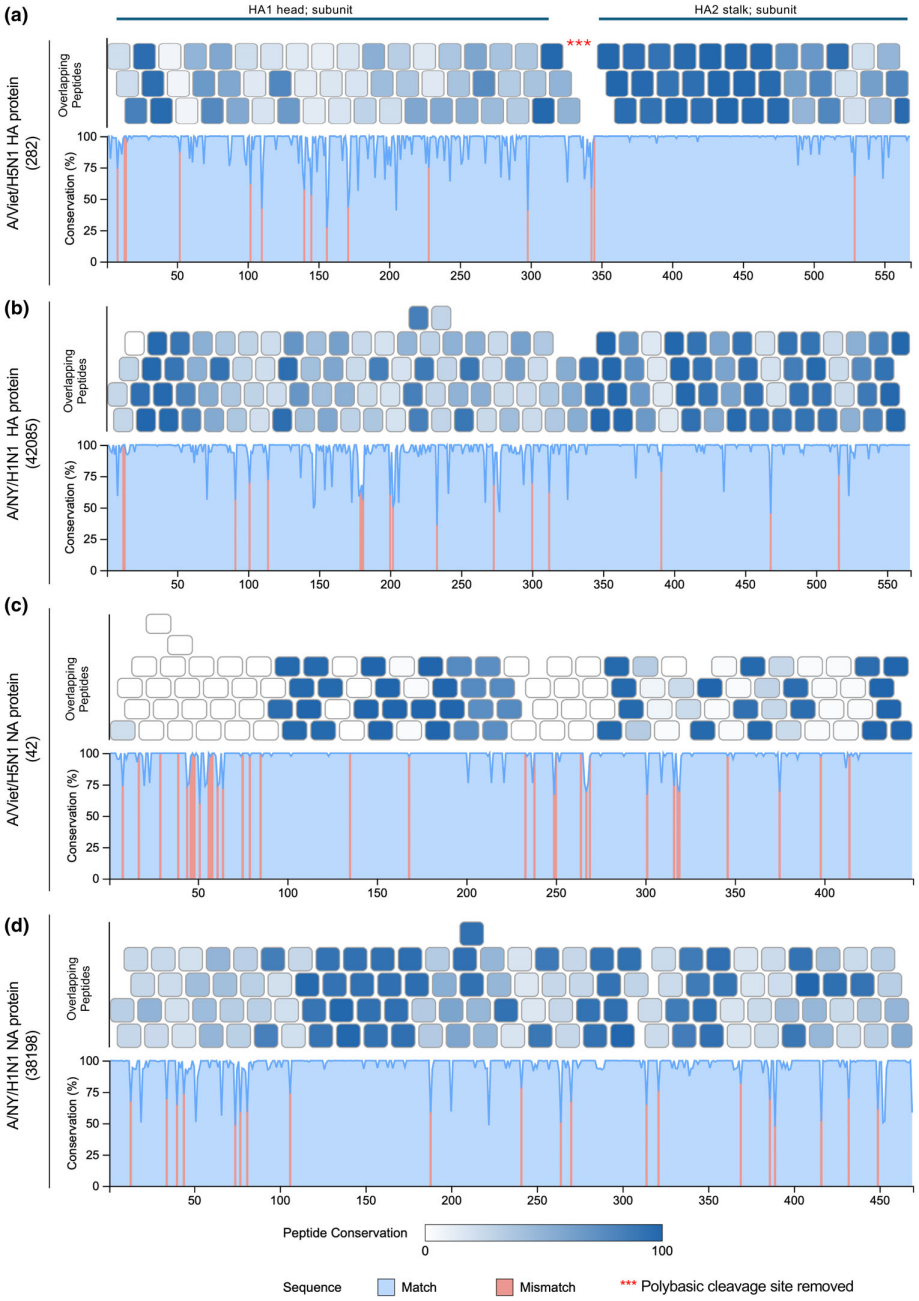
Sequence conservation of overlapping peptide pools. Comparison of overlapping peptide pools used in these experiments against a database of influenza A strains, showing **(a)** HA A/VIET/2004 against 282 H5 HA sequences, **(b)** HA A/NY/2009 against 42 085 H1 HA sequences, **(c)** NA A/VIET/2004 against 42 sequences, and **(d)** NA A/NY/2009 against 38 198 sequences. Overall conservation of the total sequences is shown in the blue histogram, indicating sites more or less prone to mutations. More conserved peptides from the overlapping peptide pool are shaded deeper blue than less conserved. Red lines indicate a mismatch from the overlapping peptide pool sequence to the most commonly observed amino acid in that location. Sequences were filtered on complete human isolates only. **(a)** Note that the overlapping HA peptides from A/VIET/2004 were generated from a modified version of the HA protein that had the HPAI polybasic cleavage site removed for safety and ethical reasons, hence there is a gap in the alignment of this peptide pool against the complete influenza H5 HA sequence.

To determine the sequence homology and conservation between the influenza viruses used in our study and the breadth of historical and current seasonal H1N1 and avian H5N1 viruses over time, full‐length human influenza A protein sequences were compared against the overlapping HA and NA peptide arrays used in our experiments (Figure 3a–d). For the HA of A/VIET/2004, the HA2 stem region was highly conserved against the breadth of human H5 sequences, in which only 8.3% (3/36) of overlapping peptides in this region contained mismatches to the consensus sequence. In contrast, the HA1 head region showed more variable conservation, with 42% (23/54) of overlapping peptides in this region containing mismatches (Figure 3a). The HA of A/NY/2009 displayed similar levels of conservation, being more conserved in the HA2 stem region while 47.5% (39/82) of peptides within the HA1 head region contained mismatches (Figure 3b). Furthermore, HA sequence alignment of the two experimental strains also highlighted the levels of residue differences, with 63% sequence identity in the HA1 head region compared to 82% in the stem region (Supplementary figure 2a).

The least‐conserved overlapping peptide array was the NA of A/VIET/2004 (Figure 3c), where 61% (67/110) of peptides contained mismatches and over half of the peptides contained no matching sequence to any of the NCBI protein sequences; although the NA database was more limited (42 sequences) compared to the HA analyses (242 sequences). In comparison, the NA of A/NY/2009 had 56 out of 115 (49%) peptides containing mismatches and all peptides had a conservation level of at least 15% (Figure 3d). NA sequence alignment of the two experimental strains was more conserved than the HA viruses, displaying 84% sequence identity (Supplementary figure 2b).

### Variable HA‐specific CD4 ^+^ T‐cell responses following group 1 peptide stimulation

**Figure 4:**
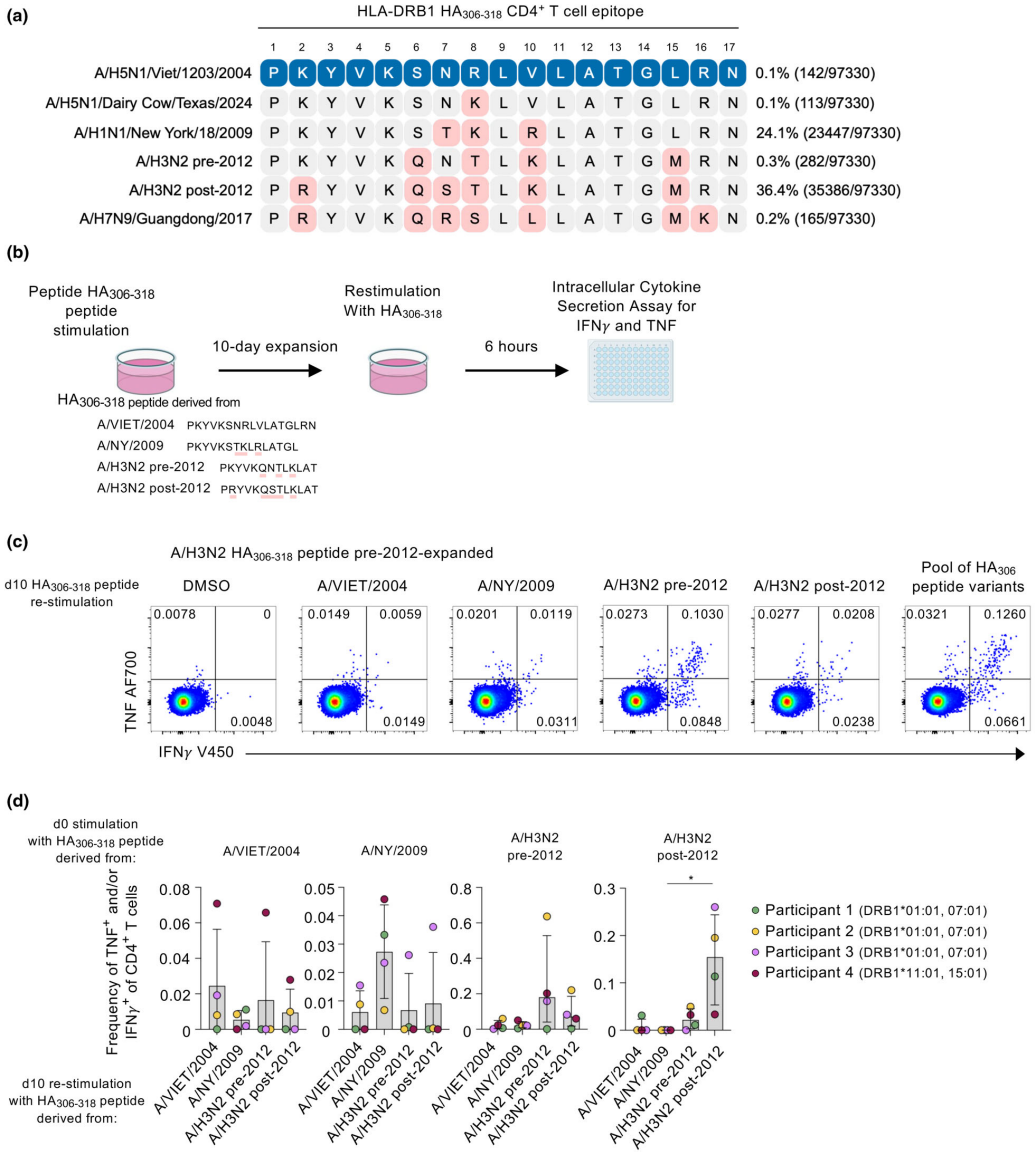
Limited cross‐reactivity between influenza A HA groups. **(a)** Sequence alignment of the HA‐PKY peptide across different seasonal and pandemic influenza A strains. Frequency of the matching sequence was compared against a database of 97 330 influenza A sequences available (NCBI Virus (captured 25/06/2025)). **(b)** Individual peptide expansion and restimulation flow diagram. **(c)** Representative staining of A/H3N2 pre‐2012 HA_306‐322_ peptide‐expanded CD4^+^ T cells after peptide restimulation and intracellular cytokine staining. **(d)** Frequency of TNF‐ and/or IFN𝛾‐positive CD4^+^ T cells from PBMCs from four healthy individuals after expansion and restimulation with either matched or mismatched HA‐PKY peptide, after DMSO background subtraction. HLA‐DRB1 typing is shown. Statistical significance determined by Friedman's test with Dunn's multiple comparisons where *P < 0.05. Bars indicate median and IQR.

We next investigated whether H1 and H5 HA‐reactive CD4^+^ T‐cell responses also extended to group 2 H3‐HA responses. To do this, we focussed on a well‐characterised and highly conserved HA_306‐318_ epitope from H3N2 viruses pre‐ and post‐2012,^19^ both variants known to be highly cross‐reactive with each other and promiscuous in generating CD4^+^ T‐cell responses from various individuals expressing either HLA‐DRB1*01:01, DRB1*04:01 or DRB1*11:01.^18^, ^19^ Therefore, PBMCs from healthy individuals expressing HLA‐DRB1*01:01 or HLA‐DRB1*11:01 were cultured with the H3N2 HA_306‐318_ peptide variants from H3N2 viruses pre‐ and post‐2012, as well as the corresponding peptide sequence from our H1 and H5 overlapping peptide pools (Figure 4a and b), before restimulating with each peptide and assessing cytokine production by CD4^+^ T cells on Day 10. Based on the sequence alignment (Figure 4a), the H5 and H1 peptides differed by three amino acid residues at Positions 7 (N→T), 8 (R→K) and 10 (V→R). The H3 pre‐ and post‐2012 peptides only differed from each other by two residues at Positions 2 (K→R) and 7 (N→S), but had up to five different residues compared to H5 and H1 peptides.

In general, cultures restimulated with their cognate peptide showed highest cytokine production compared to restimulation with alternate peptides (Figure 4c and d). The highest differences in cytokine responses were observed for the H3 peptide cultures, where responses to the alternate H3 peptide were detectable, albeit at lower levels, but minimal CD4^+^ T‐cell responses were detected towards the H1 and H5 corresponding peptide. Peptide stimulations with the H1 and H5 peptides resulted in lower cytokine responses overall; however, more comparable CD4^+^ T‐cell responses were observed towards the other peptides. However, our findings are variable and based on a small sample size and should therefore be interpreted with caution.

Overall, we provide evidence that H5N1‐reactive CD4^+^ and CD8^+^ T cells are robustly detectable in the blood of patients hospitalised with acute seasonal influenza A virus infections, at higher levels than those detected in healthy individuals for CD4^+^ T cells, along with the H1 response. Responses towards the HA of both A/VIET/2004 and A/NY/2009 strongly correlated, while NA responses were lower and weakly correlated for CD8^+^ T cells, despite higher sequence identity between the two subtypes. Our data suggest that HA is potentially more immunodominant than NA in the context of CD4^+^ T‐cell responses towards influenza virus infection. Further studies to identify highly cross‐reactive H5N1 T‐cell epitopes are warranted to determine the breadth of protection the global population may have against sustained human–human A/H5N1 transmission.

## Methods

### Participant recruitment

Patients hospitalised with influenza A viral infections were admitted to the Alfred Hospital, the Austin Hospital, or the Royal Melbourne Hospital between June 2023 and January 2026 and recruited via our well‐established DISI (Alfred HREC, #280‐14) and ISSIS (Monash HREC/15/MonH/64, Austin HREC SSA/28204/Austin‐2022, RMH HREC SSA/16/MH/280) cohorts, as previously described.^18^, ^23^ Bloods were collected at hospital admission in sodium heparin tubes and processed within 24 h. Healthy participants were recruited contemporaneously from The University of Melbourne (#31236) between June 2021 and November 2025. Informed consent was obtained from all study participants. Buffy packs were sourced from Australian Red Cross Lifeblood (2015#08) between March and April 2024. PBMCs were isolated by Ficoll‐Paque (17144003; Cytiva, Uppsala, Sweden) separation and either used fresh or cryopreserved until use. All individuals recruited were Australian residents and therefore exposure to H5Nx viruses was highly unlikely. H5Nx strains were not in circulation in Australia and environmental exposure was uncommon.

### Stimulation with overlapping peptide pools and intracellular cytokine staining

Fresh PBMCs isolated from whole blood via Ficoll–Paque separation or thawed PBMCs were plated into a 96‐well plate at 0.3–1.5e6 cells/well, as previously described.^27^ Cells were stimulated in complete‐RPMI with 100 μg/mL overlapping peptide arrays corresponding to the entire H1 HA (17 mers) and N1 NA (15 mers) of A/New York/18/2009 (H1N1)pdm09 (NR‐19245, NR‐19249; obtained through BEI Resources, NIAID, NIH, Manassas, Virginia) and the H5 HA (15 mers) and N1 NA (15 mers) A/Vietnam/1203/2004 (H5N1) (NR‐18974, NR‐19258; obtained through BEI Resources, NIAID, NIH), or 20% DMSO (1:20) as a negative control. Peptides were individually reconstituted from lyophilised powder by slowly resuspending in 100% DMSO, before diluting to 20% DMSO with Baxter Water for Irrigation (Baxter, AHF7114), and pooled. Wells were supplemented with 10 U/mL IL‐2 (11147528001; Roche, Basel, Switzerland), anti‐CD28/CD49d (1:100, 347690; BD Biosciences, Franklin Lakes, New Jersey) and Brefeldin A (1:2000; 555029; BD Biosciences) for the full 22–24 h. Following stimulation, cells were washed and stained with surface markers CD3 BV510 (317332; BioLegend, San Diego, California), CD4 BV650 (563875; BD Biosciences), CD8 PerCP‐Cy5.5 (565310; BD Biosciences) and Live/Dead NIR (L34976; Invitrogen, Waltham, Massachusetts) for 30 min on ice. Cells were washed and fixed using the BD Cytofix/Cytoperm kit (554723; BD Biosciences) according to the manufacturer's instructions, before intracellular staining with IFNγ V450 (560371; BD Biosciences), TNF AF700 (557996; BD Biosciences) and MIP‐1β ΑPC (560656; BD Biosciences). Cells were washed and resuspended in MACS buffer (PBS, 0.5% BSA, 2 mM EDTA) before acquisition on a LSRII Fortessa (BD). Data were analysed on FlowJo v10.10 (BD). Total cytokine‐producing T cells was the sum of TNF^+^ single‐positive, IFNγ^+^ single‐positive and TNF^+^IFNγ^+^ double‐positive frequencies, and values obtained from background stimulation in the DMSO control well were subtracted from peptide‐stimulated values. Any peptide stimulated value that was greater than zero after DMSO subtraction was defined as a positive response, and if less than zero was a negative response. Fresh PBMC samples were used where possible (~50% of the time) for the patient samples to preserve cell numbers for cryopreservation. For the healthy participants, almost all samples were performed from thawed PBMCs (except one individual). The source of PBMCs did not influence our results as there were no differences in the range of T‐cell responses between using fresh or thawed PBMCs from our influenza A patients.

### Influenza virus strains

Sequences used in our study included A/H5N1/Vietnam/1203/2004 (GISAID acc. number EPI25595 and EPI23047, Isolate ID EPI_ISL_4156), A/H1N1/New York/18/2009pdm09‐like (GISAID acc. number EPI177400 and EPI177405, Isolate ID EPI_ISL_29742), A/H5N1/Dairy Cow/Texas/2024 (GISAID acc. number EPI3158678 and EPI3158672, Isolate ID EPI_ISL_19014384), and A/H7N9/Guangdong/2017 (GISAID acc. number EPI960360, Isolate ID EPI_ISL_256108).

### Conservation analysis

Complete influenza A protein isolates and associated metadata were downloaded from NCBI Virus (captured 25/06/2025). The 151 718 sequences were then aligned with high fidelity using MAFFT G‐INS‐I algorithm^28^ via M3 HPC. The complete alignment was then filtered by the metadata (e.g. genotype) for downstream analysis.

### Expansion and restimulation of epitope‐specific CD4 ^+^ T cells

Thawed PBMCs were plated into a 24‐well plate at a concentration of 5e6 cells/well in complete‐RMPI supplemented with 10% human sera (HUMANABSRMC‐1; BIOIVT). Cells were stimulated with 50 μM of peptide at 37°C/5% CO_2_ before being supplemented with 10 U/mL IL‐2 after 24 h. Cells were cultured for 10 days and maintained with fresh media and IL‐2 every 2–3 days. On Day 10, cells were restimulated with 10 μM of peptide for 6 h at 37°C/5% CO_2_ plus 10 U/mL IL‐2, with Brefeldin A added for the final 4 h. After restimulation, cells were stained as described above.

### Statistical analysis

Statistical significance (*P* < 0.05, two‐tailed) was determined using the GraphPad Prism v10 software. Spearman r correlation, Mann–Whitney (unpaired analysis) and Wilcoxon (paired analysis) *t*‐tests, and Friedman's test with Dunn's multiple comparisons were used.

## Author contributions

**Lilith F Allen:** Writing – original draft; writing – review and editing; visualization; formal analysis; data curation; methodology; validation; investigation; conceptualization. **Louise C Rowntree:** Conceptualization; writing – review and editing; methodology; visualization. **Mitchell Jenzen:** Formal analysis; software; data curation; methodology; investigation; writing – review and editing. **Ruth R Hagen:** Investigation; writing – review and editing. **Nathan P Croft:** Supervision; writing – review and editing; resources. **Fiona James:** Writing – review and editing; resources. **Genevieve E Martin:** Writing – review and editing; resources. **Anthony W Purcell:** Writing – review and editing; supervision; resources. **Patricia T Illing:** Supervision; writing – review and editing; resources; formal analysis. **Steven Y C Tong:** Resources; writing – review and editing. **Allen C Cheng:** Resources; writing – review and editing. **Tom C Kotsimbos:** Writing – review and editing; resources. **Jason A Trubiano:** Resources; writing – review and editing. **Katherine Kedzierska:** Conceptualization; funding acquisition; writing – review and editing; supervision; resources; visualization; writing – original draft. **Thi H O Nguyen:** Conceptualization; funding acquisition; writing – review and editing; visualization; project administration; resources; supervision; writing – original draft.

## Conflict of interest

The authors declare no conflict of interest.

## Supporting information


Supplementary figure 1
Supplementary figure 2
Supplementary table 1

## Data Availability

All study data are included in the article.
