# Supplementary material for: CD4 + T‐cell responses directed towards A/H5N1‐derived haemagglutinin peptides increase in patients with seasonal influenza A virus infection
Source: Clin Transl Immunology. 2026 Aug 2;15(8):e70118. doi: 10.1002/cti2.70118 (PMC13429298; doi:10.1002/cti2.70118)
Supplement: Supplementary file 1 — Supplementary figure 1 Supplementary figure 2 Supplementary table 1 [file CTI2-15-e70118-s001.pdf]

SUPPLEMENTARY MATERIALS

CD4$^+$ T cell responses directed towards A/H5N1-derived haemagglutinin peptides increase in patients with seasonal influenza A virus infection

Lilith F Allen[1], Louise C Rowntree[1], Mitchell Jenzen[2,3], Ruth R Hagen[1], Nathan P Croft[2], Fiona James[4], Genevieve E Martin[1,5], Anthony W Purcell[2], Patricia T Illing[2], Steven Y C Tong[6,7], Allen C Cheng[8,9], Tom C Kotsimbos[10,11], Jason A Trubiano[4,12-14], Katherine Kedzierska*[1,15] and Thi H O Nguyen[1]*

List of Figures

Supplementary Figure 1. Participant demographics. Related to Figure 1 and 2.
Supplementary Figure 2. Sequence homology and conservation. Related to Figure 3.

List of Tables

Supplementary Table 1: Demographics of influenza patients and healthy participants used in our study. Related to Figure 1 and 2.

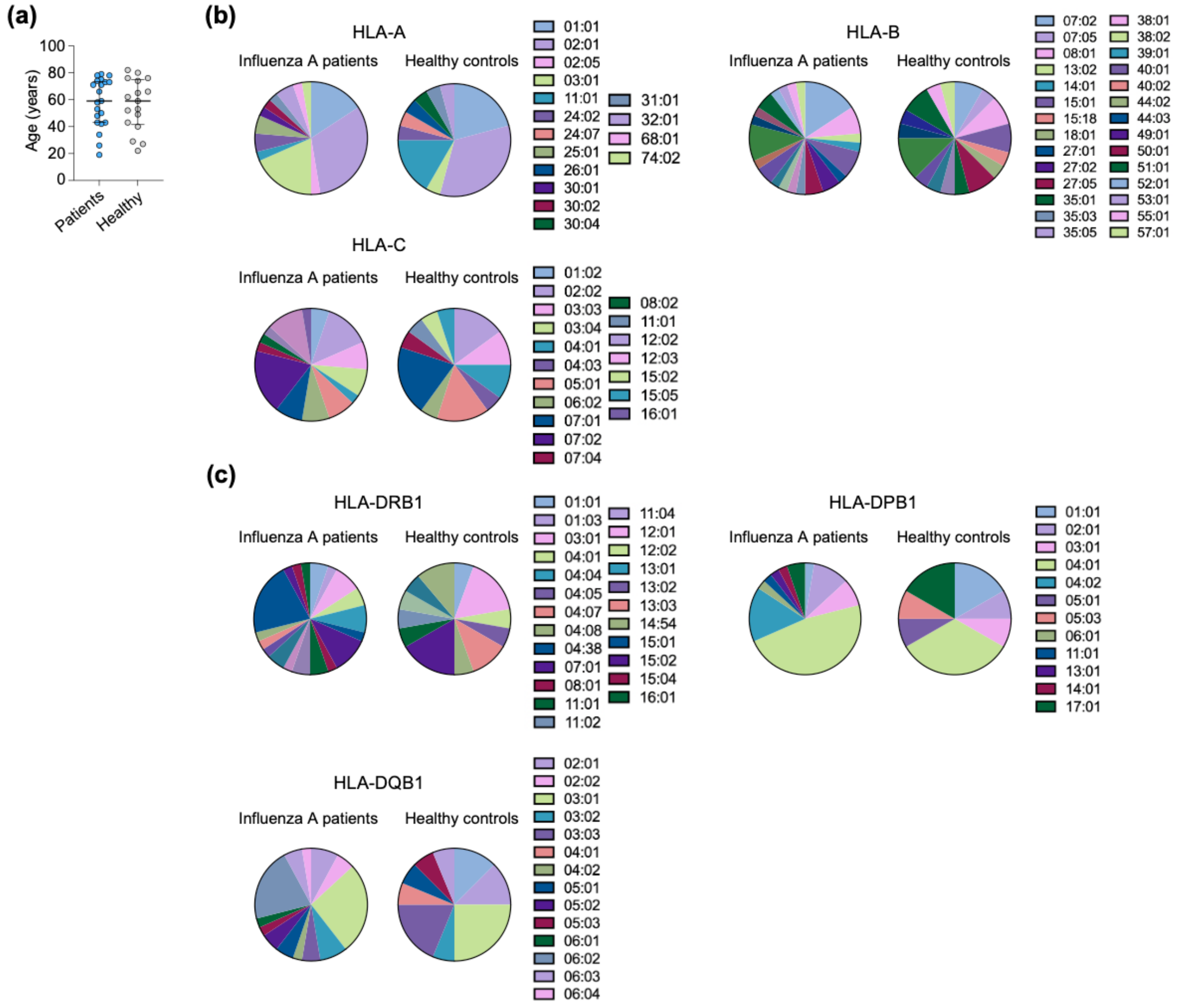

Supplementary Figure 1. Participant demographics. (a) Age distribution of influenza A-infected patients and healthy individuals. Distribution of **(b)** HLA Class I and **(c)** HLA-Class II typing that was available for influenza A-infected patients ($n = 19$) and healthy individuals (HLA-I: $n = 12$; HLA-II: $n = 9$). Each participant has 2 alleles.

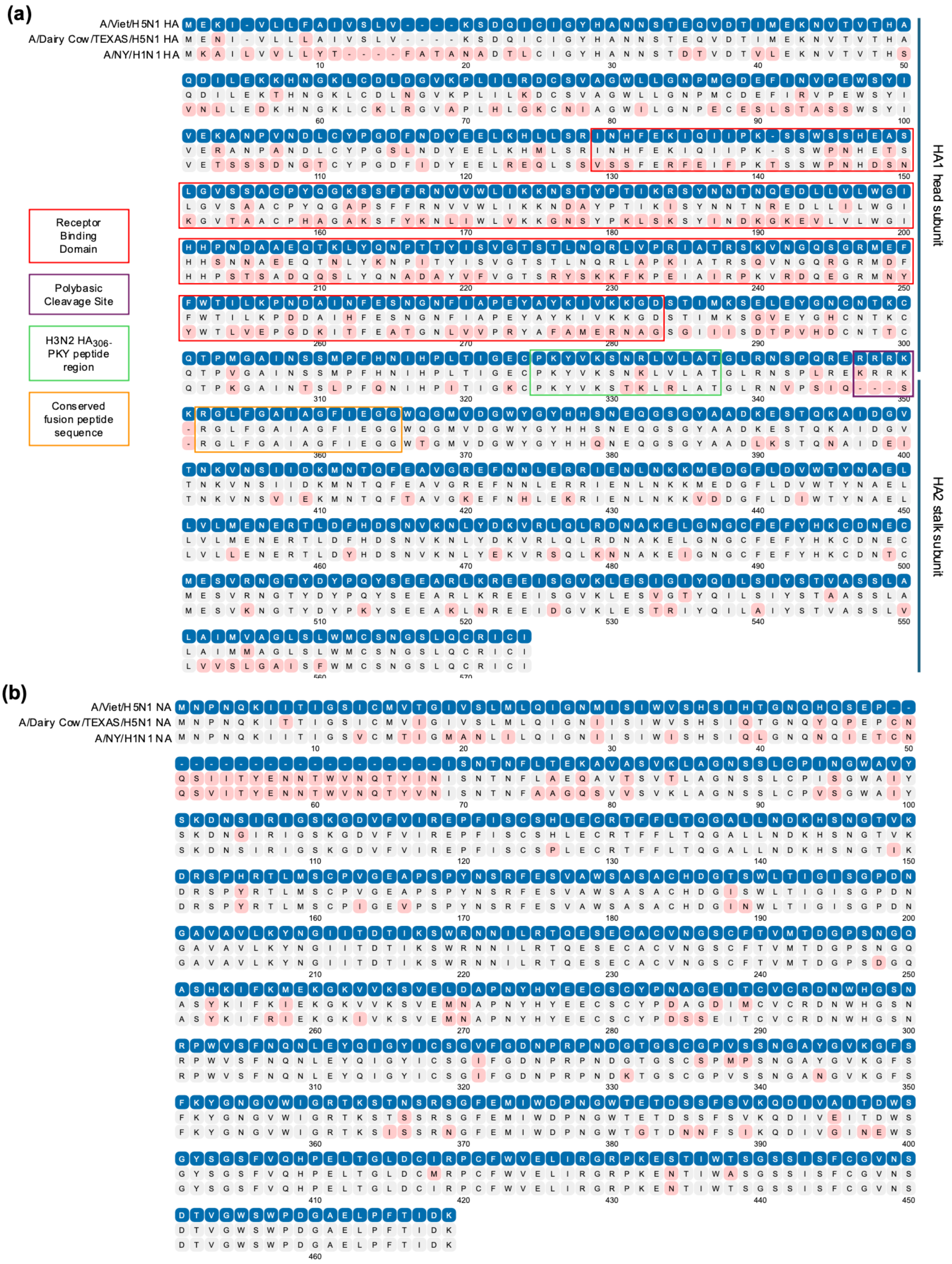

Supplementary Figure 2. Sequence homology and conservation. Full sequence comparison and multiple sequence alignment of the **(a)** HA, with key structural features highlighted and **(b)** NA of H5 A/VIET/2004, H5 A/Dairy Cow/TEXAS/2024 and H1 A/NY/2009 sequences. Mismatches are shaded red.

Supplementary Table 1: Demographics of influenza patients and healthy participants used in our study.

	**Influenza patients**	**Healthy participants**
**Number of individuals, *n***	21	17
**Age, median (range)**	59 (19-79)	59 (22-82)
**Female, *n* (%)**	8 (38)	9 (53)
**Days post symptom onset, median (range)**	4 (2-14)	-
**Birth Year**		
**Pre-1968, *n* (%)**	12 (57%)	9 (53%)
**Post-1968, *n* (%)**	9 (43%)	8 (47%)
**Diagnosis, *n* (%)**		
**Influenza A; pdmH1**	5 (24)	-
**Influenza A; H3N2**	2 (10)	-
**Influenza A; unknown**	14 (66)	-
